# Establishment of a time‐resolved immunoassay for acute kidney injury based on the detection of Kim‐1

**DOI:** 10.1002/jcla.24603

**Published:** 2022-07-23

**Authors:** Zheng Shaoxiong, Xiumei Zhou, Yuan Qin, Yu Xiaomei, Chen Lingli, Liu Xiaobin, Yigang Wang, Gong Jianguang, Shen Shuijuan, Biao Huang

**Affiliations:** ^1^ College of Life Sciences and Medicine Zhejiang Sci‐Tech University Hangzhou China; ^2^ Wuxi People's Hospital affiliated to Nanjing Medical University Wuxi China; ^3^ Zhejiang Provincial People's Hospital Hangzhou China; ^4^ Nephrology Department of Shaoxing People's Hospital Shaoxing Hospital, Zhejiang University School of Medicine Hangzhou China

**Keywords:** acute kidney injury, double‐antibody sandwich method, kidney injury molecule‐1, time‐resolved fluorescence immunoassay

## Abstract

**Aim:**

To establish a highly sensitive time‐resolved fluorescence immunoassay (TRFIA) of kidney injury molecule‐1 (Kim‐1) and evaluate its clinical value in acute kidney injury (AKI).

**Methods:**

The Kim‐1‐TRFIA was established by the double‐antibody sandwich method, and the method was evaluated. The established Kim‐1‐TRFIA was used to detect the concentration of Kim‐1 in the serum of healthy controls and patients with AKI.

**Results:**

The optimal coating antibody concentration and optimal Eu^3+^‐labeled antibody dilution ratio for Kim‐1‐TRFIA are 1 μg/ml and 1:140, respectively. The linear range is 42.71–4666.69 pg/ml. The intra‐ and inter‐assay coefficients of variation are <10%. The specificity of our Kim‐1‐TRFIA is acceptable. The recovery is between 95.14% and 102.84%. The concentration of Kim‐1 in the serum of patients with AKI is 126.50 ± 67.99 pg/ml, which is significantly higher than that in the serum of healthy controls (49.72 ± 16.40 pg/ml, *p <* 0.001). Staging patients with AKI by glomerular filtration rate shows that the serum concentration of Kim‐1 increases significantly with increasing disease severity (*p <* 0.05).

**Conclusion:**

A highly sensitive Kim‐1‐TRFIA was established. With this immunoassay, a good differential diagnosis can be made, and healthy people and AKI patients can be differentiated by detecting the concentration of Kim‐1 in the serum. Moreover, the severity of AKI patients can be determined.

## Graphical abstract

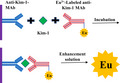

## 1 INTRODUCTION

Acute kidney injury (AKI) is part of a series of acute kidney diseases^1^ and is clinically defined as the rapid loss of renal function.^2^ At present, AKI is almost one of the most common serious diseases in clinical acute diseases. If not treated in time, AKI may be fatal.^1^ Early diagnosis and intervention can provide improved treatment options for patients with AKI, improve prognosis, and reduce mortality.^3^ AKI can be triggered by many different mechanisms, including decreased renal perfusion, obstruction of urine outflow, or exposure to nephrotoxic drugs. Regardless of the cause of stimulus, the pathogenesis of AKI is characterized by a rapid increase in serum creatinine (SCr) level and a rapid decrease in glomerular filtration rate (eGFR).^4^ However, SCr and eGFR have limitations in identifying renal function decline in a timely and accurate manner, resulting in poor sensitivity and specificity in the diagnosis of AKI.^5^ Therefore, a timely and effective AKI diagnostic and detection method is urgently needed.

In recent years, people discovered and described a variety of biomarkers, such as calprotectin, kidney injury molecule‐1 (Kim‐1), liver‐type fatty acid‐binding protein (L‐FABP), and neutrophil gelatinase‐related lipocalin (NGAL), that can indicate renal structural damage in patients with AKI. These biomarkers can be detected in urine or serum.^6^, ^7^, ^8^, ^9^ In clinics, these biomarkers, which can realize the early detection, differential diagnosis, and prognostic evaluation of AKI, are recommended as auxiliary diagnostic indicators for SCr and eGFR.^3^ At the same time, biomarkers can stratify patients, conduct targeted clinical trials, and may discover new therapeutic targets for AKI.

Kim‐1 is a type I membrane protein discovered by Ichimura et al. in 1998.^10^ Kim‐1 is expressed in the kidney and liver and is a 104 kDa peptide.^11^ In humans, proximal tubule cells are the main site of the upregulation of Kim‐1 expression.^12^ After AKI, the protein level of Kim‐1 mRNA increases substantially due to the increased expression of endogenous tubule cells.^13^ In addition, Kim‐1, a phosphatidylserine receptor, can recognize apoptotic cells and guide them to enter the lysosome when the kidney undergoes acute injury.^14^ Kim‐1 also acts as a receptor for oxidized lipoproteins and can help phagocytes recognize the “eat me” signal of apoptotic cells,^15^ which may be because Kim‐1 plays a role in regulating the immune response in AKI. Kim‐1 enhances the clearance of apoptotic cells by surviving tubule cells, thereby downregulating the proinflammatory immune response. Therefore, Kim‐1 plays an important role in the occurrence and the related repair process of AKI. A review of literature (until November 2019) published by Jiwen Geng et al. shows that Kim‐1 is a good predictor of AKI in adults; it has high sensitivity and specificity.^1^ Although relevant studies increased in recent years, the clinical application of Kim‐1 in the early diagnosis of AKI requires experimental support and clinical research.

Currently, enzyme‐linked immunosorbent assay (ELISA) and electrochemiluminescence immunoassay (ECLIA) are commonly used to detect the concentration of Kim‐1 in serum or urine.^16^, ^17^, ^18^ However, ELISA uses macromolecular enzymes to label antibodies, which can easily change the original conformation of the antibody or block the binding site of the antibody, resulting in a decrease in its activity.^19^ In addition, ECLIA's instruments are expensive, and the measurement cost is high. Moreover, ECLIA is not suitable for the high‐throughput detection of large samples.^20^ Therefore, the establishment of a new serological detection method to detect this promising biomarker can provide value for further clinical applications.

**FIGURE 1:**
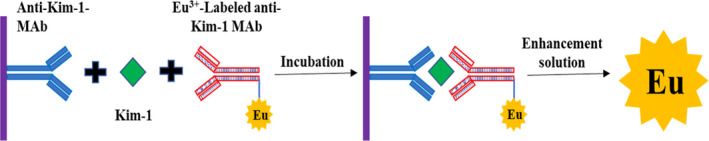
Experimental principle of the Kim‐1‐TRFIA method

This study aims to establish a highly sensitive time‐resolved fluorescence immunoassay (TRFIA) to detect the serum Kim‐1 concentration of healthy controls and patients with AKI to provide a reference for the clinical diagnosis and treatment of patients with AKI. The experimental principle is shown in Figure 1.

## 2 MATERIALS AND METHODS

### 2.1 Reagents and Instruments

Tween‐20, bovine serum albumin (BSA), and Proclin‐300 were purchased from Sigma‐Aldrich (USA). An Eu^3+^‐labeled kit, anti‐Kim‐1 antibody (coating antibody, labeled antibody, mouse McAb), and Kim‐1 antigen were purchased from Zhejiang Bosch Biotechnology Co., Ltd. All other chemicals were of analytical grade. The ultrapure water used in the whole experiment was prepared using the Barnstead water purifier (Thermo Fisher Scientific, USA). The Sephadex G‐50 chromatography column was purchased from Shanghai Xibao Biotechnology Co., Ltd. The automatic time‐resolved fluorescence immunoassay analyzer DR6608 was purchased from Guangdong Foshan Daan Medical Equipment Co., Ltd. The Biofuge‐fresco (D‐37520 Osteroded) was purchased from Kendro Lab Products.

### 2.2 Buffers

The coating buffer was 50 mmol/L Na_2_CO_3_–NaHCO_3_ (pH 9.6). The assay buffer was 50 mmol/L Tris–HCl (pH 7.8) containing 8 mmol/L NaCl, 0.1% BSA, 50 μmol/L DTPA, 0.1 ml/L Tween‐20, 0.1% NaN_3_, and 40 μg/ml HBR 1. The washing solution was a buffer solution containing 0.48 g/L Tris, 12.49 g/L NaCl, and 1.11 g/L Tween‐20 adjusted to pH 7.8 by hydrochloric acid. The enhancement solution contained glacial acetic acid with a volume concentration of 3.6‰, 0.5 g/L sodium acetate, 0.05 g/L β‐naphthoyl trifluoroacetone, 0.03 g/L trioctyl phosphine oxide, and a volume concentration of 1 ‰ Triton X‐100 mixed solution. The labeling buffer was 50 mmol/L Na_2_CO_3_ (pH 9.0). The elution buffer was 50 mmol/L Tris–HCl containing 0.9% NaCl, 0.05% Proclin‐300, 0.02% Tween‐20, and 0.2% BSA (pH 7.8). The blocking solution was 50 mmol/L Tris–HCl containing 0.9% NaCl, 1% BSA, and 0.05% NaN_3_ (pH 7.8).

### 2.3 Serum samples

Blood samples were collected from healthy controls (*n* = 78), patients with AKI (*n* = 22) from Wuxi People's Hospital (Jiangsu Province, China). The sample was centrifuged at 1200 *g* for 5 min to obtain serum and stored at −20°C. The preparation of serum samples did not include hemolysis and lipid turbidity steps. The selection criteria for the healthy control group were as follows: no history of kidney disease, autoimmune disease, or human immunodeficiency virus; hepatitis B virus; and hepatitis C virus infection. All patients with AKI were diagnosed through the hospital's urine routine, urine sedimentation, urine biochemistry, and pathological diagnosis. According to the internationally accepted staging standard of glomerular filtration rate, the 22 patients with AKI were divided into five groups, as follows: G1 stage (≥90 ml/min/1.73 m^2^), G2 stage (60–89 ml/min/1.73 m^2^), G3 stage (30–59 ml/min/1.73 m^2^), G4 stage (15–29 ml/min/1.73 m^2^), and G5 stage (<15 ml/min/1.73 m^2^). All registered subjects provided written informed consent and agreed to participate in this study. The subject was approved by Wuxi People's Hospital affiliated with Nanjing Medical University.

### 2.4 Method of Kim‐1‐TRFIA

#### 2.4.1 Preparation of solid‐phase antibody

The Kim‐1 coating antibody was diluted to 1 μg/ml with coating buffer. Then, 100 μl of Kim‐1 coating antibody was added into a 96‐well plate at 4°C overnight. The antibody was washed once and added with 150 μl blocking solution. After blocking for 2 h at room temperature, the blocking solution was discarded, and the antibody was dried under vacuum, sealed, and stored at −20°C for later use.

#### 2.4.2 Preparation of labeled antibody

The Kim‐1 labeled antibody (0.3 mg) was placed into a 50 kD Ultracel ultrafiltration tube and centrifuged at 10,000 *g* for 6 min, and the filtrate was discarded. The antibody was added with 300 μl labeling buffer. The mixture was centrifuged eight times at 10,000 *g* for 6 min, added with 50 μl labeling buffer to the ultrafiltration tube, inverted, and centrifuged twice at 3000 *g* for 1 min to collect the filtrate. After ultrafiltration, 0.06 mg Eu^3+^ chelate was added to the Kim‐1‐labeled antibody, and the reaction was shaken overnight at 28°C. On the second day, the reaction solution was subjected to chromatography through the column, and the Sephadex G‐50 was first equilibrated with the elution buffer. The reaction solution was added to the Sephadex G‐50 for chromatography, and the eluate was collected. The fluorescence value of the eluate was determined. Part of the first eluate with high fluorescence value was collected, freeze‐dried in vacuum, and stored at −20°C.

#### 2.4.3 Preparation of Kim‐1 standards

The high‐concentration Kim‐1 antigen was diluted with assay buffer to obtain standard concentrations of 23.31, 116.69, 233.31, 1173.69, 2333.31, and 4666.69 pg/ml, and each solution was stored at 2–8°C.

#### 2.4.4 Kim‐1‐TRFIA experimental procedure

The sample to be tested (50 μl) and 50 μl Eu^3+^–Kim‐1‐McAb were added into a 96‐well plate coated with Kim‐1 antibody and reacted with shaking at 37°C for 1 h. The mixture was washed six times with a cleaning solution and added with 100 μl enhancement solution to dissociate Eu^3+^ on Eu^3+^–Kim‐1‐McAb. DR6608 was used to measure the fluorescence of the sample. The concentration of the sample was determined in accordance with the standard curve of Kim‐1.

#### 2.4.5 Optimization of the best coating antibody concentration

The Kim‐1‐coated antibody (5 mg/ml) was diluted with coating buffer to obtain concentrations of 0.25, 0.5, 1, 2, and 4 μg/ml; then, it was coated overnight at 4°C. After blocking, the antibody was mixed with 50 μl of 1173.69 pg/mL antigen standard. The fluorescence value at the different coating concentrations was detected in accordance with the experimental procedure. The most appropriate coating concentration was selected.

#### 2.4.6 Optimization of the optimal dilution ratio of Eu^3+^–Kim‐1‐McAb

Eu^3+^–Kim‐1‐McAb was diluted at 1:35, 1:70, 1:140, 1:280, 1:560, and 1:1020 with assay buffer. The antigen at 0 concentration point and 1173.69 pg/ml were added to the coated plate and added with Eu^3+^–Kim‐1‐McAb at a certain dilution ratio in accordance with the experimental procedure. The fluorescence under different dilution ratios was detected, and the most appropriate dilution ratio was selected.

#### 2.4.7 Optimization of optimal response time

Add 1173.69 pg/ml antigen standard to the Kim‐1 coated antibody plate, then add a certain ratio of diluted Kim‐1 labeled antibody solution, set 6 different reaction times, respectively, 20, 40, 60, 80, 100, and 120 min. After incubation at 37°C in a constant temperature incubator, the fluorescence values measured at different reaction times were detected, and the most appropriate reaction time was selected.

### 2.5 Kim‐1‐TRFIA Methodological Evaluation

#### 2.5.1 LOD, LOQ and linearity

The fluorescence of Kim‐1 standard was determined at different concentrations (23.31, 116.69, 233.31, 1173.69, 2333.31, and 4666.69 pg/ml), and a standard curve was drawn using the prism. The *R* ^2^ of the linear equation was used as a linear indicator. The fluorescence value at the 0 concentration point was measured 10 times, then the Limit of Detection (LOD), Limit of Quantification (LOQ) of the method, was calculated in accordance with the ACS Committee on Environmental Improvement (LOD = S_b_ + 3σ, LOQ = S_b_ + 10σ, where S_b_ is the mean of the blank signal measurements and σ is the standard deviation of the blank signal measurements).^21^

#### 2.5.2 Precision

Kim‐1 antigen standards (233.31, 1173.69, and 2333.31 pg/mL) were collected. Ten independent experiments were performed on these three concentrations of standards to obtain intra‐ and inter‐assay coefficients of variation (*CV*s).

#### 2.5.3 Specificity

L‐FABP, NGAL, and Cys C, which could also be used as indicators of kidney damage, were used as potential interference antigens to assess specificity. This method was used to conduct 10 independent experiments on these three indicators.

#### 2.5.4 Recovery

Separately add the known low, medium, and high concentration nephropathy patient serum to a known concentration standard at a ratio of 9:1. Three repeated experiments were performed, and the recovery rate was determined in accordance with the following formula: recovery rate (%) = (Determined Concentration/Theoretical Concentration) × 100%.

#### 2.5.5 Correlation between Kim‐1‐TRFIA and ELISA

To investigate the reliability of the Kim‐1‐TRFIA method, we measured the Kim‐1 concentrations in these 22 clinical serum samples simultaneously with a purchased commercial ELISA kit and compared their correlations.

### 2.6 Clinical application of Kim‐1‐TRFIA

The newly established Kim‐1‐TRFIA detected the serum samples of patients with AKI and healthy controls to evaluate the clinical diagnostic value of Kim‐1‐TRFIA in patients with AKI.

### 2.7 Statistical analysis

SPSS 13.0 (Chicago, USA) was used for statistical mean and standard deviation (*SD*). The *mean* ± *SD* was used for the statistics of normally distributed data, and the median (interquartile range) was used for non‐normally distributed data. The t‐test was used to analyze the significant difference between the two groups of data. The intra‐assay precision and inter‐assay precision were expressed as the coefficient of variation (*CV*). Graphs were made using GraphPad Prism 16.1 (San Diego, USA). The Jonckheere‐Terpstra test was used to compare the Kim‐1 concentrations and various parameters in different GFR stages in AKI patients. ROC curve analysis was used to determine the diagnostic value of Kim‐1 in AKI patients. All *p* values were two‐tailed, and the test standard was *p* = 0.05; *p* < 0.05 indicated that the statistical results are significantly different.

## 3 RESULTS

### 3.1 Optimization of Kim‐1 coating antibody concentration

**FIGURE 2:**
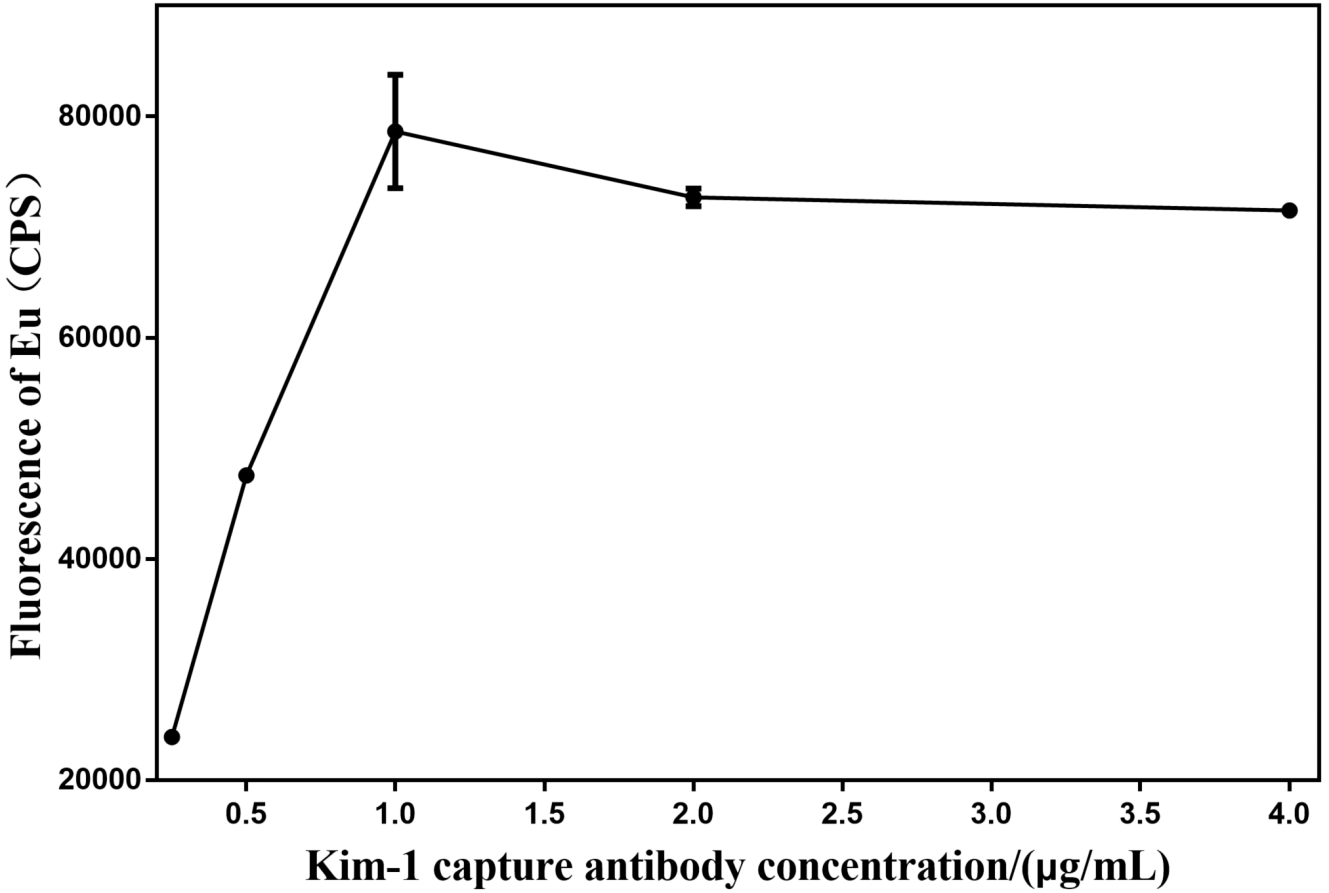
Optimization of Kim‐1 coating concentration

As shown in Figure 2, with increasing concentration of Kim‐1 coated antibody, the fluorescence curve of 1173.69 pg/ml standard sample showed an upward trend, and it reached the highest point at 1 μg/ml. Therefore, the optimal concentration of Kim‐1‐TRFIA‐coated antibody was 1 μg/ml.

### 3.2 Optimization of the dilution ratio of Eu^3+^–Kim‐1‐McAb

**FIGURE 3:**
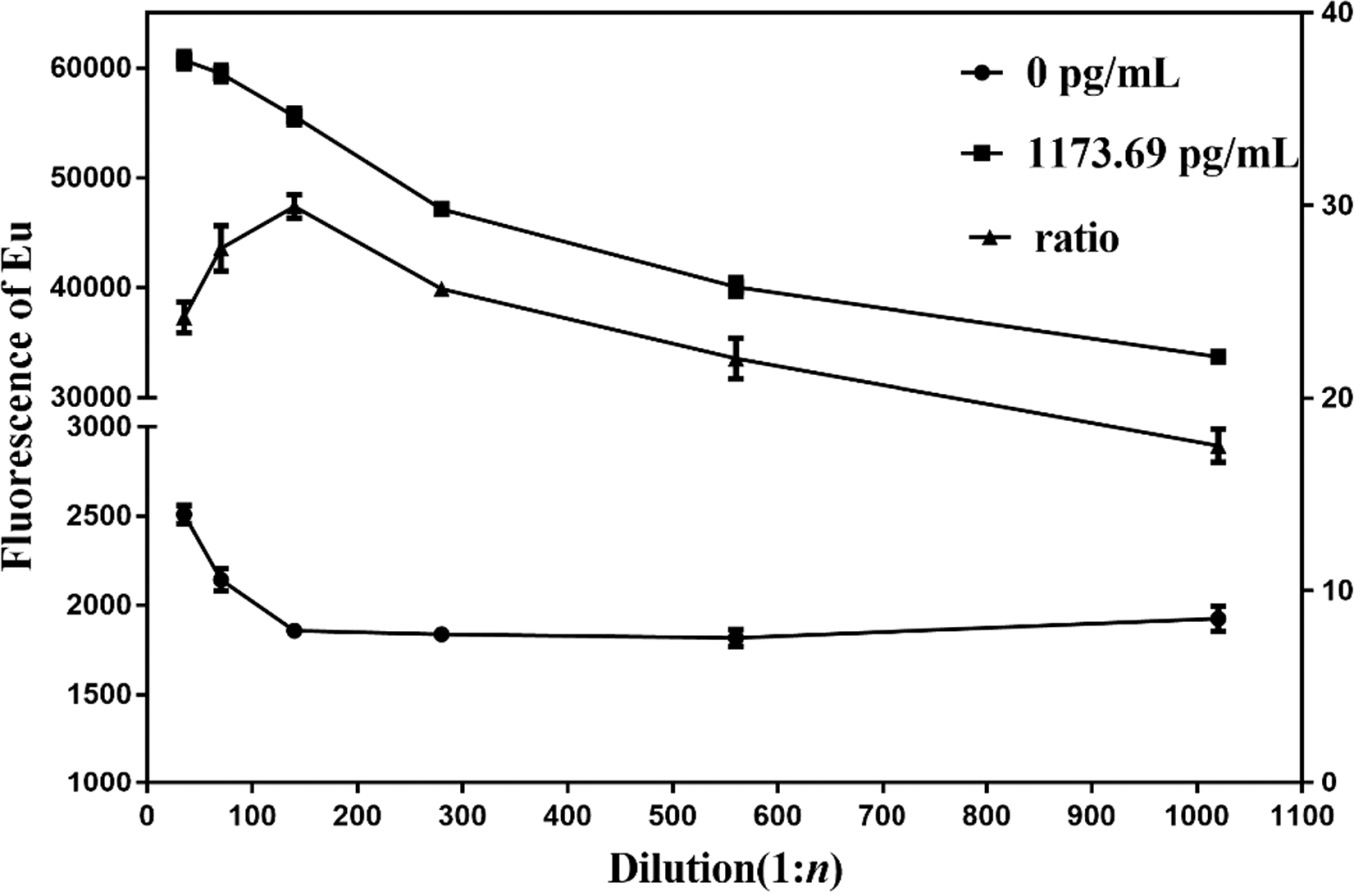
Optimization of the dilution ratios of Eu^3+^–Kim‐1‐McAb

As shown in Figure 3, in the curve of the 1173.69 pg/ml concentration point, as the dilution ratio increases, fluorescence gradually decreases. Compared with the curve at the 0 concentration point, when the dilution ratio is 1:140, the fluorescence ratio of Kim‐1‐TRFIA is highest. Therefore, 1:140 is chosen as the best dilution ratio of Eu^3+^–Kim‐1‐McAb for Kim‐1‐TRFIA.

### 3.3 Optimization of optimal response time

**FIGURE 4:**
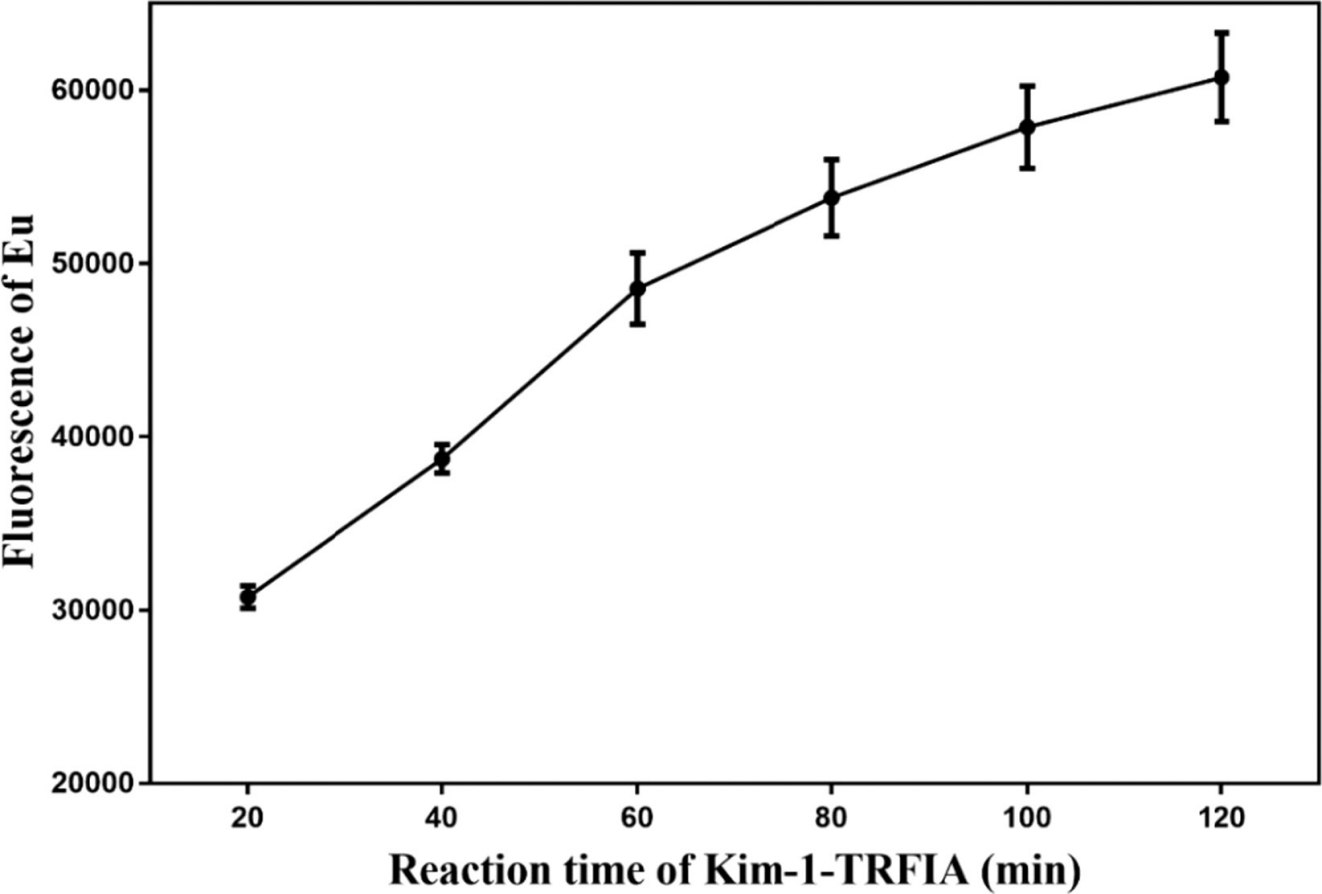
Optimization of Kim‐1‐TRFIA reaction time

As shown in Figure 4, with increasing Kim‐1‐TRFIA reaction time, the fluorescence value curve corresponding to the 1173.69 pg/ml antigen standard gradually increased and then became flat after 80 min of reaction. Therefore, we chose 80 min as the optimal reaction time for Kim‐1‐TRFIA.

### 3.4 Methodological evaluation of Kim‐1‐TRFIA

#### 3.4.1 LOD, LOQ and linearity

**FIGURE 5:**
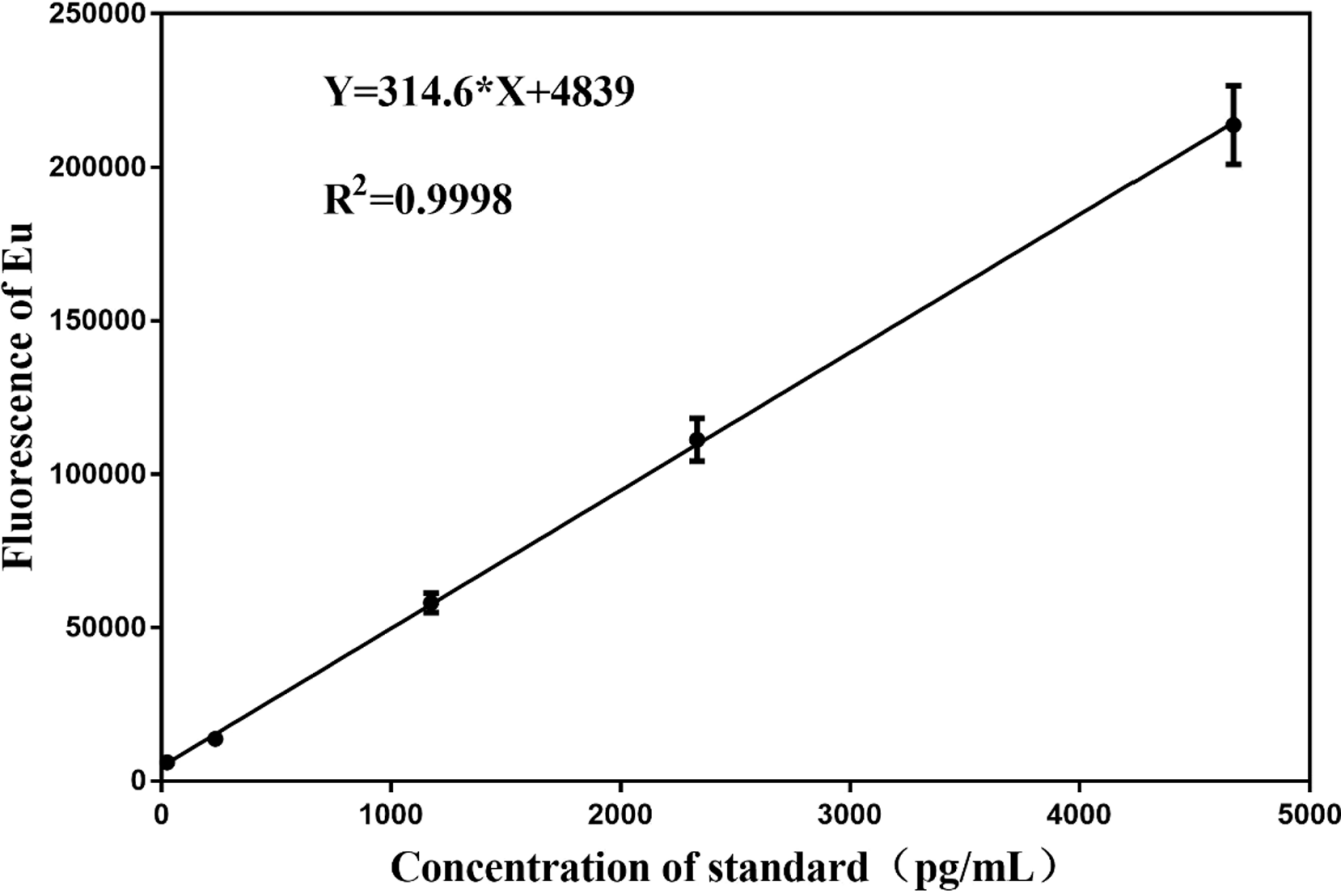
Standard curve of the Kim‐1 standard

As shown in Figure 5, Kim‐1‐TRFIA has a good linear relationship between 23.31 and 4666.69 pg/ml (*R* ^*2*^ = 0.993). The LOD and LOQ were 16.31 and 42.71 pg/ml, respectively. Therefore, the linear range of Kim‐1‐TRFIA is 42.71–4666.69 pg/ml (Figure 5).

#### 3.4.2 Precision

As shown in Table 1, precision is expressed using *CV*. Intra‐ and inter‐assay *CV*s are <10%, which shows that the precision of the method is acceptable.

**TABLE 1:** Precision of Kim‐1‐TRFIA

	Concentration (pg/ml)	Average (pg/ml)	Standard deviation	CV (%)
Inter‐assay (*n* = 10)	233.31	250.95	14.15	5.64
	1173.69	1143.84	48.46	4.24
	2333.31	2267.85	98.54	4.35
Intra‐assay (*n* = 10)	233.31	244.65	8.40	3.43
	1173.69	1123.65	56.12	4.99
	2333.31	2215.57	150.15	6.78

#### 3.4.3 Specificity

Table 2 shows that the cross‐reactivity of L‐FABP, NGAL, and Cys C are 0.001%, 0.001%, and 0.001%, respectively. This result indicates that Kim‐1‐TRFIA has no cross‐reaction with these three biomarkers.

**TABLE 2:** Cross‐reactivities of Kim‐1‐TRFIA

Interferent	Concentration (ng/ml)	Determined (ng/ml)	Cross‐reactivity (%)
Kim‐1	1.169	1.148	98.20
L‐FABP	1000	0.014	0.001
NGAL	2000	0.021	0.001
Cys C	5000	0.035	0.001

#### 3.4.4 Recovery

Table 3 shows that the average recoveries of Kim‐1‐TRFIA are 95.14%, 95.52%, and 102.84%, respectively, indicating that this experiment is not interfered by the serum.

**TABLE 3:** Recoveries of Kim‐1‐TRFIA

Samples	Theoretical concentration (pg/ml)	Measured (pg/ml)	Recovery (%)
S1	863.31	821.31	95.14
S2	243.81	232.89	95.52
S3	369.81	380.31	102.84

#### 3.4.5 Correlation between Kim‐1‐TRFIA and ELISA

**FIGURE 6:**
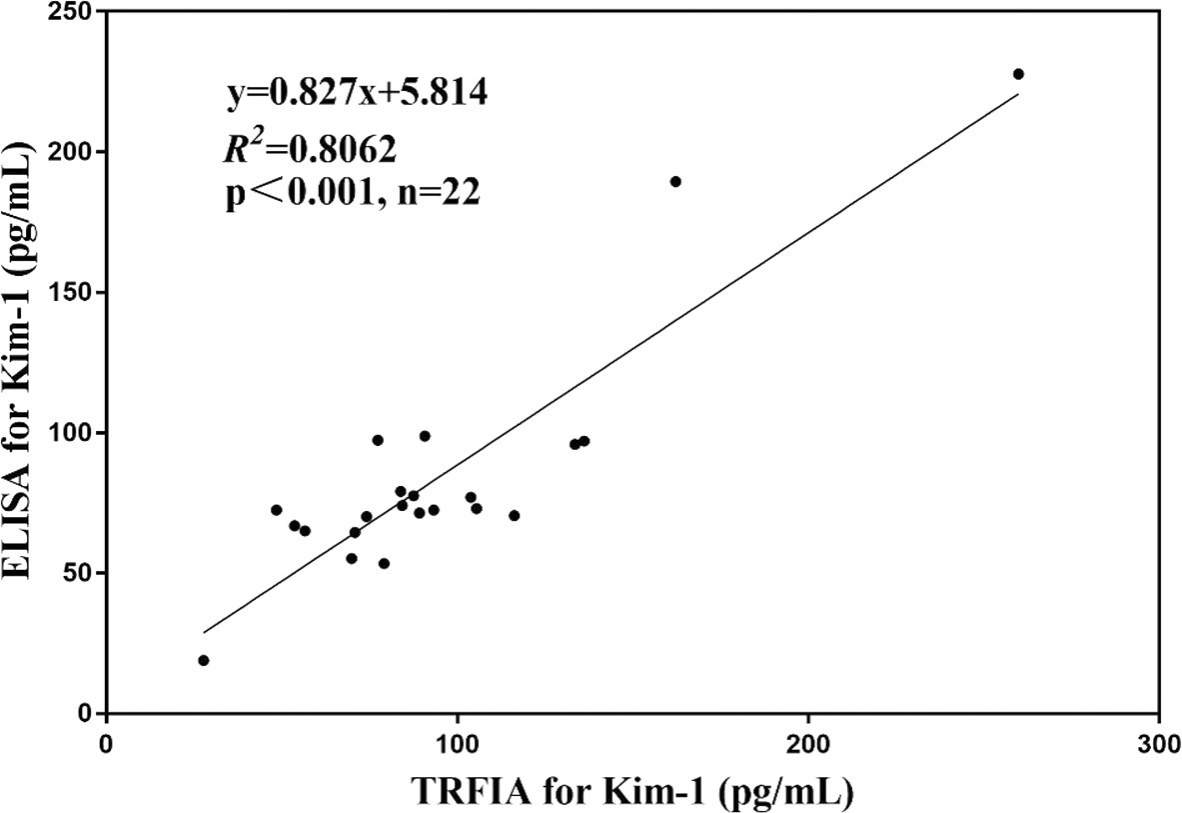
Correlation of Kim‐1 concentration results between ELISA and the newly established Kim‐1‐TRFIA

ELISA and TRFIA were used to detect the concentrations of Kim‐1 in the serum of these 22 patients with AKI. The correlation coefficient of the two methods was 0.8062, thereby indicating that the detection results of the two methods had a high correlation (Figure 6).

### 3.5 Clinical application of Kim‐1‐TRFIA

**FIGURE 7:**
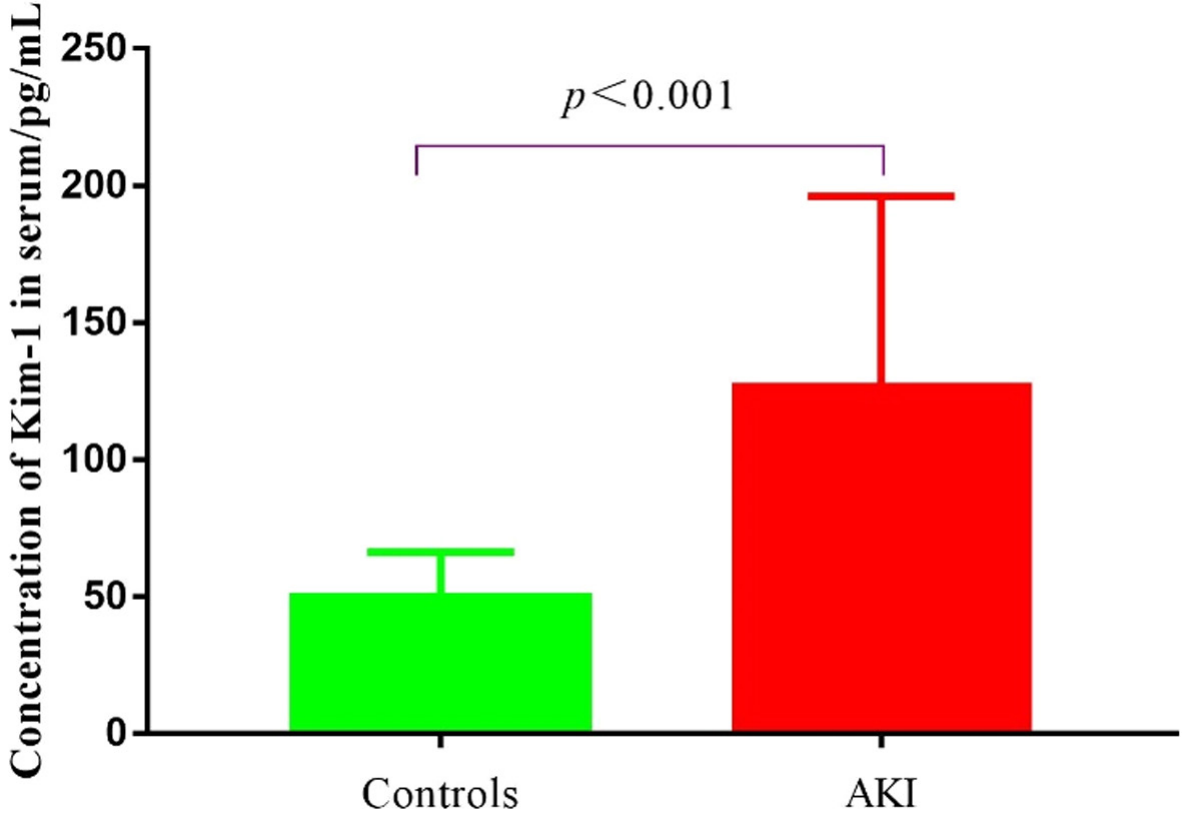
Kim‐1 concentration in patients with AKI patients and healthy controls

We used 78 healthy volunteers as the normal control group for these 22 AKI patients. The concentration of Kim‐1 in these 100 samples was determined by Kim‐1‐TRFIA. The concentration range of the control group was 49.72 ± 16.40 pg/ml, and the *mean* + 2*SD* was 82.52 pg/ml. Therefore, we set the concentration of Kim‐1 in normal human serum at <82.52 pg/ml. The serum concentration of Kim‐1 in AKI patients was significantly higher than those in normal controls (126.50 ± 67.99 pg/ml vs. 49.72 ± 16.40 pg/mL, *p* < 0.001, Figure 7).

**FIGURE 8:**
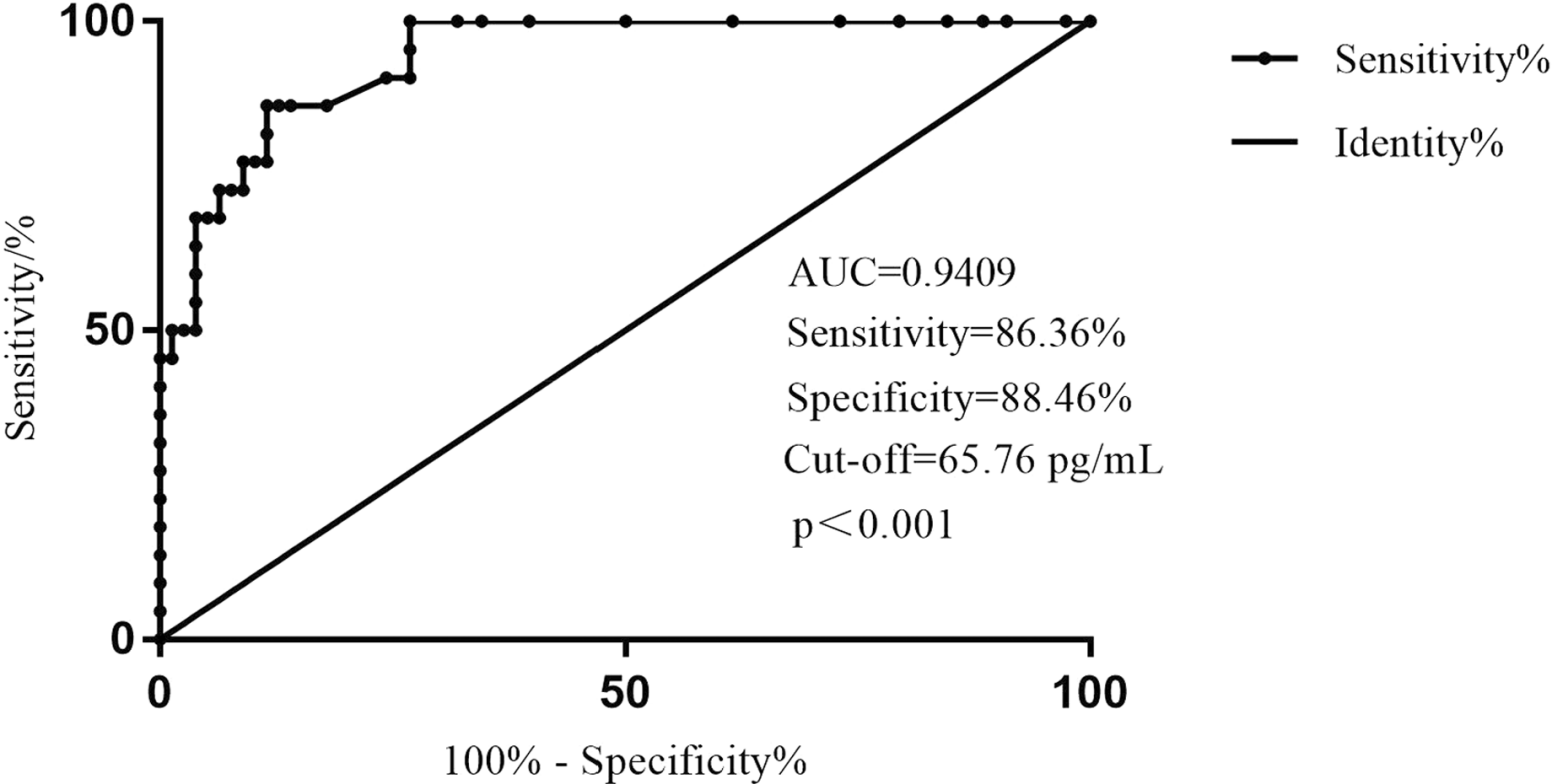
ROC curves of normal controls and AKI patients

To evaluate the clinical diagnostic value of serum Kim‐1 concentration in patients with AKI, we compared the patients with AKI with the normal control group and drew the ROC curve. As shown in Figure 8, the area under the curve (AUC) was AUC = 0.9409, *p* < 0.001. When the cut‐off was 65.76 pg/mL, the sensitivity and specificity were 86.36% and 88.46%, respectively. These results indicated that the concentration of serum Kim‐1 had high application value in the differential diagnosis of AKI patients and normal controls.

We divided 22 patients with AKI into 5 stages by eGFR, compared the data of various indicators of patients with AKI at different stages, and conducted Jonckheere‐Terpstra test analysis. As shown in Table 4, UREA (*p* = 0.015) and CREA (*p* < 0.001) increased significantly with the progression of disease from G1 to G5, and eGFR (*p* < 0.001) decreased significantly with the progression of disease. The serum concentration of Kim‐1 was also significantly increased in patients with AKI at different stages (*p* = 0.016).

**TABLE 4:** Comparison of parameters in patients with AKI at different eGFR stages

Parameters	G1‐G2	G3	G4‐G5	Total	Jonckheere‐Terpstra
Number (male/female)	7 (3/4)	11 (5/6)	4 (4/0)	22(12/10)	0.448
Age (years)	58.57 ± 23.35	54.73 ± 13.75	62.00 ± 15.41	57.27 ± 17.86	0.779
UREA (mmol/L)	6.34 ± 1.46	7.84 ± 3.26	23.90 ± 11.65	10.29 ± 8.50	0.012
CREA (μmol/L)	81.86 ± 10.31	134.41 ± 27.22	375.58 ± 38.45	161.54 ± 106.73	<0.001
eGFR (ml/min/1.73 m^2^)	80.13 ± 18.91	45.59 ± 8.92	11.50 ± 4.90	50.38 ± 26.94	<0.001
URIC (μmol/L)	311.06 ± 199.13	241.49 ± 103.51	568.20 ± 318.52	323.03 ± 225.21	0.306
Kim‐1 (pg/ml)	86.33 ± 30.33	130.67 ± 60.67	182.00 ± 86.33	126.00 ± 70.00	0.028

## 4 DISCUSSION

Many studies confirmed that Kim‐1 is a potential marker of AKI and can be used for the early diagnosis of AKI.^22^ However, due to the lack of commercial trials, the application of serum Kim‐1 in the diagnosis of clinical AKI is limited. In the past few years, several methods for detecting Kim‐1 have been established. Studies on ECLIA based on the biotin–avidin amplification system is used to detect the level of Kim‐1 in human urine. During the analysis, streptavidin and biotin specifically bind to form a double‐antibody sandwich structure. Although this method can detect multiple indicators at once, the detection steps of this method are cumbersome, and the detection time is long (5 h).^16^ Studies established ELISA to detect the level of Kim‐1 in human urine, but similarly, the detection steps of ELISA are cumbersome and require long detection time (4.5 h).^17^ At the same time, the sensitivity of ELISA is low, leading to many false negative results. Insufficient stability and detection range are also common shortcomings of this method.^23^ Therefore, a sensitive and fast detection method is needed.

TRFIA is an emerging technology with a lanthanide chelate as a marker. TRFIA has high sensitivity (10^−18^ mol/L), simple operation, easy automation, and wide standard curve range and is not subject to natural fluorescence interference from samples. TRFIA has the many features, such as simple preparation, stable, and no radioactive contamination.^24^ In addition, lanthanides have high specific activity as a marker, ideal stability, and minimal effect on biological activity.^25^ The addition of the dissociation enhancement buffer in the measurement phase can increase the initial fluorescence intensity by a million times.^26^

In this study, we established Kim‐1‐TRFIA for the first time to detect the concentration of Kim‐1 in human serum. This method could detect the concentration of Kim‐1 in the serum with only 80 min of reaction, which could gain valuable time for clinical diagnosis and treatment of AKI. The LOD and LOQ of Kim‐1‐TRFIA were 16.31 and 42.71 pg/mL, respectively, which was higher than those of many immunoassays for quantitative Kim‐1 concentration.^17^, ^27^, ^28^ The extremely low cross‐reactivities observed showed that Kim‐1‐TRFIA had high specificity for Kim‐1. The good correlation between Kim‐1‐TRFIA and ELISA kit indicates the reliability of this method.

Studies applied Kim‐1 for the early detection of AKI and found that Kim‐1 as a biomarker of AKI shows a moderate recognition ability (AUCs = 0.69–0.7).^29^ At the same time, Nickolas TL et al.^30^ explored the role of Kim‐1 in predicting persistent AKI and performed well (AUC = 0.74). In this study, the area under ROC curve of serum Kim‐1 concentration in AKI patients and healthy control group was 0.9409, close to 1, which has a better diagnostic reference value compared with the above two studies.

In the current study, patients with specific Kim‐1 values were distinguished from those with AKI, but changes in Kim‐1 values with the progression of kidney disease could not be determined.^1^ The serum concentration of Kim‐1 in 22 patients with AKI staging according to eGFR decreased significantly from G1 to G5 (*p* < 0.05). So, the serum concentration of Kim‐1 was closely related to the severity of kidney injury. This finding allowed the more accurate evaluation of the diagnostic value of Kim‐1, and the concentrations of Kim‐1 at different stages of nephropathy had a very important clinical guiding significance for emergency and serious patients.^7^

Kim‐1‐TRFIA has high accuracy and sensitivity and is easy to automate. Kim‐1‐TRFIA is a valuable human serum Kim‐1 detection system. However, the study had several limitations. First, all serum samples were from kidney patients in China, and so, the results may not apply to other ethnic groups. In addition, the sample size of patients with AKI was small. We should further increase the sample size to conduct more repeated experiments that can verify the reliability of our results. In addition, the study used a cross‐sectional design rather than a longitudinal observation. This study is expected to provide an ideal alternative method for the prediction and prognosis of patients with AKI.

## FUNDING INFORMATION

Funding was provided by the Social Development Fund of Zhejiang Province (No. LGF20H200008), Key Research and Development Project of Zhejiang Province (No. 2020C03066), the National Natural Science Foundation of China (No. 82172336), Key Research and Development Project of Hangzhou (No. 202004A23), Key Research and Development Project of Hangzhou (No. 202004A23).

## Data Availability

All the data that related to this study are available from the corresponding author upon reasonable request.
